# Prevalence and association of polycystic ovary syndrome and hidradenitis suppurativa in underrepresented groups

**DOI:** 10.1007/s00403-024-02971-9

**Published:** 2024-06-08

**Authors:** Peter Y. Ch’en, Michelle Toker, Gloria Chen, H. Dean Hosgood, Kristina L. Campton, Steven R. Cohen

**Affiliations:** 1 Division of Dermatology, Department of Medicine, Albert Einstein College of Medicine, Bronx, NY USA; 2 Yale School of Medicine, New Haven, CT USA; 3 Department of Dermatology, Weill Cornell Medical College, 1305 York Avenue 9F, New York, NY 10022 USA

**Keywords:** Hidradenitis suppurativa, Polycystic ovary syndrome, PCOS, Social determinants of health, Socioeconomic status

## Abstract

Hidradenitis suppurativa (HS) is an inflammatory disorder of follicular biology; androgens are believed to be involved in its pathogenesis. Polycystic ovary syndrome (PCOS) is similarly characterized by hyperandrogenism. Previous studies have found a lasting association of HS and PCOS. Socioeconomic status (SES) has been described as a comorbidity for both HS and PCOS that has not been accounted for in prior studies; we sought to investigate this association while adjusting for this. We also analyzed the prevalence of PCOS among HS patients. Using the *All of Us* database, female HS patients were stratified by PCOS diagnosis and compared by age, race, and ethnicity. Female HS patients were also nearest-neighbor propensity-score matched to controls at a 4:1 ratio, selecting for race, ethnicity, age, ever smoker, alcohol use disorder, obesity, type II diabetes, Medicaid status, and community deprivation index. Univariable and multivariable logistic regression was conducted to estimate the effect of HS on the presence of PCOS. The distribution of race among HS patients with PCOS was significantly different than HS patients without PCOS. A total of 1,022 female HS patients and 4,088 matched female controls were included. Significantly more patients carried a diagnosis of PCOS compared to controls (8.8% versus 4.3%, *p* < .001). In multivariable logistic regression, PCOS was significantly associated with HS [OR 1.71 (95% CI 1.34–2.17)]. This is the first study investigating the association of HS and PCOS within the *All of Us* database. We found that females with HS had a 1.34- to 2.17-fold increased odds of having PCOS, which is consistent with previous analyses. However, our analysis, in addition to controlling for common medical co-morbidities found in both HS and PCOS, also accounts for markers of SES at an individual and community level, further strengthening the association of HS with PCOS.

## Introduction

Hidradenitis suppurativa (HS) is an inflammatory disorder of follicular biology primarily affecting the intertriginous skin regions. It manifests as painful pustules, nodules, abscesses, sinus tracts, and scarring. Androgens are believed to be involved in its pathogenesis [1]. 

Polycystic ovary syndrome (PCOS) is similarly characterized by hyperandrogenism and manifests with irregular menses and ovulatory dysfunction. PCOS has been shown to be more prevalent among individuals with HS; and, previous studies found a stable association after adjusting for shared comorbidities [1]. 

Differences in prevalence of PCOS between racial groups among patients with HS is not well established. In addition, socioeconomic status (SES), a comorbidity for both HS and PCOS, has not been accounted for in prior studies investigating the link between the two conditions [2–4]. We sought to investigate the prevalence of PCOS among HS patients and the association of PCOS and HS, adjusting for SES as a potential confounder. Insurance status and community deprivation index (census tract-based score of material deprivation) regarding individual and area SES were used as metrics to explore this association [5]. The NIH *All of Us* database aims to enroll a large, diverse cohort of the US population from traditionally underrepresented groups in research and was used for this study.

## Materials and methods

A total of 409,420 participants were part of the *All of Us* database at the time of our analysis. Females with hidradenitis suppurativa were identified using International Classification of Diseases (ICD)-10-L73.2 or Systemized Nomenclature of Medicine (SNOMED) 4,241,223/59,393,003 billing and documentation codes. Females with PCOS were identified using ICD-10-E28.2 or SNOMED 40,443,308/237,055,002.

Female HS patients were stratified by PCOS diagnosis and compared by age, race, and ethnicity using Student’s t-test and Pearson’s chi-squared.

Female HS patients were then nearest-neighbor propensity-score matched (i.e., female patients without HS) at a 4:1 ratio, according to race, ethnicity, age, ever smoker status, alcohol use disorder, obesity, type II diabetes, non-Medicaid status, and community deprivation index. Pearson’s chi-squared, Fisher’s exact, and Student’s t-tests were used to assess differences between the HS cohort and matched controls.

Multivariable logistic regression was conducted to estimate HS effect(s) on PCOS.

## Results

Of 99/1,073 (9.2%) female HS patients with PCOS, 31.3% were Black, 62.6% were White, and < 20 were Other (Table 1); this distribution varied significantly from those without PCOS (*p* < .01).

**Table 1:** Prevalence of PCOS among female patients with HS

Variable	Female HS Patients without PCOS (*n* = 974)	Female HS Patients with PCOS (*n* = 99)	*p*-value
Age, years [mean (SD)]	47.73 (14.07)	36.77 (8.66)	< 0.001***
*Race (%)*			0.006**
Black or African American	467 (47.9)	31 (31.3)	
Other	51 (5.2)	< 20^†^	
White	456 (46.8)	62 (62.6)	
*Ethnicity* = Not Hispanic or Latino (%)	938 (96.3)	94 (94.9)	0.693

^†^Per the All of Us Research Program data privacy policy, variables with less than 20 participants are noted as < 20 to protect individual participant data

** *p* < .01

*** *p* < .001

A total of 1,022 female HS patients and 4,088 matched female controls with complete data were included in the matched analysis. Significantly more female HS patients had PCOS (8.8% versus 4.3%, *p* < .001) (Table 2). In multivariable logistic regression, PCOS was significantly associated with HS [OR 1.71 (95% CI 1.34–2.17)]. Markers of SES did not affect this relationship.

**Table 2:** Female HS patients versus matched female controls

Variable	Matched Female Controls^‡^ (*n* = 4088)	Female HS Patients (*n* = 1022)	*p*-value
Age, years [mean (SD)]	48.28 (14.95)	48.14 (14.75)	0.785
*Race (%)*			0.359
Asian	< 20^†^	< 20^†^	
Black or African American	1849 (45.2)	446 (43.6)	
Middle Eastern or North African	28 (0.7)	< 20^†^	
More than one population	132 (3.2)	42 (4.1)	
White	2064 (50.5)	519 (50.8)	
*Ethnicity =* Not Hispanic or Latino (%)	3971 (97.1)	982 (96.1)	0.101
Ever Smoker (≥ 100 cigarettes in lifetime)	2011 (49.2)	503 (49.2)	1
Alcohol Use Disorder	398 (9.7)	101 (9.9)	0.934
Obesity	2983 (73.0)	745 (72.9)	0.868
Type II Diabetes Mellitus (T2DM)	1654 (40.5)	420 (41.1)	0.738
Non-Medicaid Insurance (%)^††^	2282 (55.8)	585 (57.2)	0.434
Community Deprivation Index [mean (SD)]	0.33 (0.06)	0.33 (0.06)	0.603
PCOS	199 (4.9)	90 (8.8)	< 0.001***

^‡^Matched controls selected via 4:1 nearest neighbor propensity score matching without replacement with co-variates of race, ethnicity, age, ever smoker, alcohol use disorder, T2DM, insurance status, and community deprivation index that yielded adequate balance ≤ 0.06

^†^Per the *All of Us* Research Program data privacy policy, variables with less than 20 participants are noted as < 20 to protect individual participant data

^††^No insurance/Medicaid versus non-Medicaid insurance serving as a proxy for low SES

***p < .001

## Discussion

This is the first study of prevalence and association of HS and PCOS within the *All of Us* database. We found HS patients with PCOS were more likely to be White; contrary to current prevalence studies of PCOS reporting similarity between Black and White people [6, 7]. The *All of Us* database overrepresents a diverse population, while previous studies include predominantly Caucasian populations. Further nationally representative prevalence studies are needed to investigate this potential discrepancy, which may influence screening and treatment guidelines.

We also found that females with HS had a 1.71-fold increased odds of having PCOS (95% CI: 1.34–2.17), which aligns with previous analyses [1, 7]. However, we also account for markers of SES at an individual and community level. Considering this potential confounder, our work further strengthens the association of HS with PCOS. Future studies should include measures of SES, as HS disproportionately affects those of lower SES [4]. 

While the shared pathophysiological mechanism between HS and PCOS is currently unknown, meta-inflammation – a chronic proinflammatory state – may play a role [8, 9]. Meta-inflammation may arise from obesity, a known comorbidity of both HS and PCOS; excess adipose tissue can lead to the upregulation of proinflammatory molecules that may play a role in the manifestation of the two diseases.

Study limitations include retrospective design and the use of diagnostic codes to identify cases of HS and PCOS. The diagnostic criteria for HS and PCOS that is marked by these codes may vary by provider. Data from the *All of Us* database may not be generalizable to the United States population given its intentional overrepresentation of minority groups. However, our use of propensity score matching and comparisons within the database allows us to make valid inferences.

This study highlights the need for broader prevalence studies and corroborates the postulated association of HS and PCOS in the literature when controlling for a wide range of medical comorbidities. Further investigation of the underlying mechanism, clinical implications, and the role of hormonal therapies are warranted.

## Data Availability

Research data supporting the results of the manuscript can be found in the publicly accessible NIH All of Us database.
